# Psychological burden of quarantine in children and adolescents: A rapid systematic review and proposed solutions

**DOI:** 10.12669/pjms.36.5.3088

**Published:** 2020

**Authors:** Nazish Imran, Irum Aamer, Muhammad Imran Sharif, Zubair Hassan Bodla, Sadiq Naveed

**Affiliations:** 1 Nazish Imran, MBBS; FRCPsych (London); MRCPsych (London); MHPE. Associate Professor, Department of Child and Family Psychiatry, King Edward Medical University/Mayo Hospital, Lahore, Pakistan; 2 Irum Aamer, MBBS; FCPS. Senior Registrar, Academic Department of Psychiatry and Behavioral Sciences, King Edward Medical University/Mayo Hospital, Lahore, Pakistan; 3 Muhammad Imran Sharif, MBBS; FCPS. Senior Registrar, Department of Child and Family Psychiatry, King Edward Medical University/Mayo Hospital, Lahore, Pakistan; 4 Zubair Hassan Bodla, MBBS. Medical Officer, Department of Child and Family Psychiatry, King Edward Medical University/Mayo Hospital, Lahore, Pakistan; 5 Sadiq Naveed, MD; MPH. Assistant Professor of Psychiatry and Behavioral Sciences, University of Kansas Medical Center, Kansas, USA.

**Keywords:** COVID-19, Children, Adolescents, Quarantine, Mental health, Stigma

## Abstract

As COVID-19 grips the world, many people are quarantined or isolated resulting in adverse consequences for the mental health of youth. This rapid review takes into account the impact of quarantine on mental health of children and adolescents, and proposes measures to improve psychological outcomes of isolation. Three electronic databases including PubMed, Scopus, and ISI Web of Science were searched. Two independent reviewers performed title and abstract screening followed by full-text screening. This review article included 10 studies. The seven studies before onset of COVID 19 about psychological impact of quarantine in children have reported isolation, social exclusion stigma and fear among the children. The most common diagnoses were acute stress disorder, adjustment disorder, grief, and post-traumatic stress disorder. Three studies during the COVID-19 pandemic reported restlessness, irritability, anxiety, clinginess and inattention with increased screen time in children during quarantine. These adverse consequences can be tackled through carefully formulated multilevel interventions.

## INTRODUCTION

Children and adolescents account for 42% of the world’s population with 26% being younger than 15 years of age.^1^ Initial studies suggest that although children and adolescents are less likely to be infected with COVID-19 and they stay asymptomatic or have milder symptoms of illness if get infected, but they are not indifferent to the psychological distress of pandemic.^2^ Children aged 2 years are reported to be aware of the changes around them.^3^ Uncertainties regarding pandemic itself, strict social distancing measures, widespread and prolonged school closures, parental stressors, and loss of loved ones are likely to affect children and adolescent’s wellbeing in addition to specific psychological effects of quarantine and isolation.^4^,^5^

The word “quarantine” originated from the Italian words “quaranta giorni,” which mean 40 days.^6^ Quarantine is a state of enforced isolation of people with exposure to a contagious disease to prevent the spread of illness.^6^ Quarantine and isolation have been used as disease containment measures in Leprosy, Plague, Severe Acute Respiratory Syndrome (SARS), Middle East Respiratory Syndrome (MERS), Ebola, and more recently in COVID-19.^7^-^9^ Citywide quarantine measures are being imposed around the world to prevent the transmission of COVID-19 in the communities. Furthermore, people with infection including children and adolescents are either being isolated at homes or in state run isolation facilities as per different countries’s policies.

Quarantine and isolation are no doubt an unpleasant and distressing experience for all people who face it.^7^,^10^ Uncertainty of disease status, restrictions on mobility and daily activities, separation from loved ones, and boredom may contribute to negative effects of quarantine.^7^ Literature suggests significant psychological issues in quarantined individuals including anxiety, depression, sleep difficulties, anger and post-traumatic stress disorder in addition to suicide in adult.^11^-^13^ Duration of quarantine, provision of inadequate information, boredom and frustration, fears about being infected, financial losses, and stigma were some of the factors identified with stress in quarantined population.^7^ Stigma in particular has been a recurrent theme in literature with regard to distress associated with quarantine.^14^-^17^

There is lack of conclusive evidence of the impact of quarantine and isolation on children and adolescents. Routines, social interactions and friendships are among the most important factors responsible for children’s normal psychological development. Being quarantined or isolated often break their usual routines and can make an already challenging situation far more difficult for all children and adolescents, particularly for those with special needs or preexisting psychiatric difficulties.^5^ Since the COVID-19 outbreak related disease containment measures and school closure has become relevant to all affected countries around the globe, urgent evidence synthesis is needed to help policy makers understand the mental health outcomes of quarantine in children and adolescents. The World Health Organization recommends rapid reviews in such situations due to urgency of this matter.^18^ In view of the scarce information about the mental health implications of quarantine in younger individuals, we undertook a review of evidence to explore quarantine’s likely effects on stigma, children and adolescent’s mental health and psychological wellbeing, and factors that contribute to or mitigate these effects.

## METHODS

This rapid review was conducted according to PRISMA guidelines. Three electronic databases including PubMed, Scopus, and ISI Web of Science were searched using following search terms:

(Stigma OR stigmas OR stigmatization) AND (psych* OR Mental OR Anxiety OR Depression OR Stress OR Insomnia OR adjustment) AND (quarantine* OR Patient isolation OR isolate* OR lockdown OR lock-down OR Cordon) AND (Child* OR Adolescent OR Adolescence OR Youth)

Two independent reviewers performed the title and abstracts screening, followed by the screening of full texts and discrepancies were resolved through discussion. Manual search of included full-text articles was performed. The authors also propose interventions to reduce distress from these disease containment measures.

### Eligibility Criteria

### Our inclusion criteria were

1. Studies including primary research
2. English-only articles
3. Studies including data on the prevalence of mental illness or psychological wellbeing or stigma, or on factors associated with mental illness or psychological wellbeing (ie, any predictors of psychological wellbeing during or after quarantine).
4. Age<18 years.

### Our exclusion criteria were

1. Studies that were not evaluating for psychological impact and stigma related to quarantine in children and adolescents.
2. Unreliable data sets, duplicate, overlapping, or non-peer-reviewed articles.
3. Review articles, research articles without available full texts, book chapters, conference papers, theses, case reports and case series, abstract-only articles, and animal studies.

### Data extraction

Descriptive statistics regarding study population, country of study, scales used to measure for outcome, summary of results, and limitations were extracted. Two independent reviewers extracted the data of included articles and discrepancies were resolved through discussion.

## RESULTS

**Fig.1:**
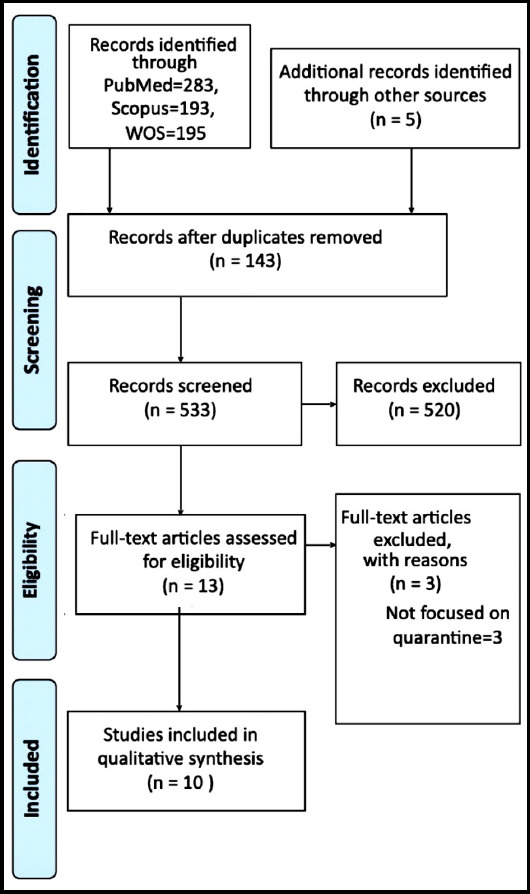


The initial literature search revealed 530 unique citations, among which 10 studies were included after the screening process. Fig.1 elaborates the screening process in PRISMA flow diagram and Table-I provides a summary of included studies.

**Table-I:** Summary of Included Articles.

Study	Study population	Country of study	Name of disaster	Reporter of symptoms	Scales used to measure primary outcome	Summary of results	Limitations
Franco et al, 2007 ID 8	Family members of Ventilated Children Age range: 1.8-19 years. Gender distribution: Not mentioned	Canada	Children requiring ventilation at home	Parents and siblings	Home visit, observation, interviews	-Increase risk of social exclusion, isolation, and social sufferings. -Parents were the protective figures for their child.	None reported
Dyb et al, 2011 ID 7	Children 319 Age range: 6-18 years Gender distribution:53 % females	Norway	Tsunami, South East Asia, 2004	Parents	CSDC, IES-parental PTSD	-Three to four traumatic tsunami related incidences among children and parents with a significant associations between children’s and parents’ exposure to the stressor and parents’ PTSD with children’s levels of posttraumatic stress reactions. -About 98% of the parents had a PTSD reaction.	-Informational bias -Correlational findings and study cannot prove causality.
Sprang et al, 2013 ID 2	398 parents of children Age range: Parents 18-67 yrs, age of children not mentioned Gender Distribution: Female=78%	USA, Canada, Mexico	Influenza A virus subtype H1N1 pandemic, SARS, Avian influenza	Parents	UCLA PTSD-RI- Parent Version and, PCL-C	-Increased risk of PTSD among quarantined children with service utilization of 34%. -Most common diagnoses were acute stress disorder (16.7%), adjustment disorder (16.7%), and grief (16.7%). Only 6.2 of these children were diagnosed with PTSD.	-Recall bias. -Parent’s subjective perception -Lack of generalizability.
Santavirta et al, 2015 ID 3	Children evacuated to temporary foster care Age range: 38-78 years Gender distribution Women: 22021 Men: 23442	Finland	World War II	Census and Hospital records	No scales were used. Hospital records were used to confirm reasons of admission	-Evacuation was not a significant predictor of admission to psychiatric hospital. -Evacuated females experienced more mood disorders ( 2.19% vs. 1.10% )	- Absence of formal test of statistical power for within sibling results. - Late follow-up for participants - Lack of consideration for the time of the child spend in Sweden and socioeconomic status of foster families.
Denis-Ramirez et al, 2016 ID 10	24 children orphaned by Ebola Age range: 8-14 years. Gender distribution: not mentioned	Sierra Leone (West Africa)	EBOLA virus epidemic	Close informants from NGO’s and social service	Drawings and captions	-Stigma, disease fear, and health campaigns were frequently described in the drawing.	-Reporting bias -Influence of each other’s drawings. -Social desirability bias -Active epidemic outbreak
Elsbernd et al, 2018 ID 11	Nine cancer survivors Age range:15-25 yrs. Gender distribution**:** seven females	Denmark	Cancer	Self	Semi-structured interviews	-Systemic and social challenges due to symptoms and inability to return to school. -Lack of official resources. -Cancer survivors stated that they felt that their peers did not understand their situation.	-Small sample size -Information bias, -Author’s self-selection bias -Lack of generalizability
Muenks et al, 2018 ID 12	Children with MRSA skin and soft tissue infection Age range of index patient: 1-17 years Gender of index patients: 54% females	USA	MRSA	Primary caregivers	13 question qualitative interview	-42% reported a change in household interactions. -The majority (91%) of participants shared their child’s MRSA diagnosis with people outside of their household.	-Lack of standardized tools -Lack of baseline knowledge
Jiao et al, 2020 ID 13	320 children Age range: 3-18 years Gender distribution: 168 girls, 142 boys	China	COVID 19	Parents	Online survey based on DSM-5, cross cultural assessment of anxiety and depression.	-Increased fear of contracting infection, inattention, persistent inquiry, clinging, and irritability -Child distress was relieved by use of entertainment through social media and physical exercise.	Preliminary report
Orgilés et al, 2020 ID 6	Children Age range: 3-18 years Gender distribution: Female=48%	Italy, Spain	COVID-19	Parents	A survey with four sections, a) Sociodemographic of parents b) parental perception of emotional effects of c) parent's perception of family coexistence d) children’s routines: time of screen use, physical activity, and hours of sleep during quarantine compared to before COVID-19.	-Change in emotional state and behaviors with symptoms such as difficulty concentrating (77%), boredom, irritability, restlessness, nervousness, feelings of loneliness, being more uneasy and more worried. -12% of the Italian and Spanish parents informed that family coexistence was difficult or very difficult, these parents tended to report their children more restless, angry and irritable. -Increased use of screen time, less physical activity, and increased sleep.	Online survey with generalizability of study Children’s perceptive was not taken Differences in rates of occurrences of emotional distress among children of different nationalities could be because of different rules of lock down and severity of spread of covid-19.
Pisano et al, 2020 ID 5	Children Age range: 4-10 years Gender distribution: not mentioned	Italy	COVID-19	Parents	12-item ad-hoc questionnaire including three areas (four questions for each domain: Regressive, Oppositional behaviors, and adaptation behaviors	-Irritability, regressive symptoms, and being listless to the activities they had done before the pandemic (one in two). -Adaptive capabilities were exhibited more among 8-9 years. old children.	-Online survey, did not assess if parent filled form separately for each child or answered in general for all children. -Parent’s response about pandemic was not assessed. -Possible sampling errors because of an online research -Technical skills of parents -Limited number of questions in the survey.

CSDC=Child Stress Disorders Checklist, DSM=Diagnostic and Statistical Manual of Mental Disorders, IES-R=Impact of Event Scale- Revised, MRSA=Methicillin-resistant Staphylococcus aureus, NGO=Non-governmental organization, PCL-C=PTSD CheckList – Civilian Version, PTSD=Posttraumatic Stress Disorder, UCLA PTSD-RI=UCLA PTSD Reaction Index, SARS=Severe Acute Respiratory Syndrome, SSTI=Skin and soft tissue infections, USA=United States of America.

### Study Designs and Scales Used

Among the included studies, study design was cohort in four studies, cross-sectional in three, and descriptive qualitative in three. Outcome measures were assessed by using surveys (n=4), interviews (n=4), focus groups (n=1), review of hospital records (n=1), home visits and observations (n=1), drawing and captions (n=1). These scales have been summarized in Table-I. Parents or caregivers were reporter of symptoms in eight studies whereas one study used hospital records.^19^ and other used drawings and captions by children.^20^

### Nature of Disaster

Disease or disaster containment measures were adapted due to COVID-19 in three studies.^21^-^23^ Other disasters included Influenza A virus subtype H1N1 pandemic, Severe Acute Respiratory Syndrome (SARS), and Avian Influenza in one study^13^ followed by World War II^19^, Tsunami^24^, children requiring mechanical ventilation^25^, Ebola^26^, cancer survivors^20^, and Methicillin-resistant Staphylococcus aureus infection of skin and soft tissues.^27^

### Summary of Included Articles

A qualitative study was performed in children requiring mechanical ventilation that highlights the importance of sociological framework in improving our understanding of the medical and social problems.^25^ It also describes socialization processes that can help resolve the social exclusion, isolation and social sufferings experienced by disabled children and their families. In most cases, parents were working as a protective capsule for their disabled child by creating their own social norms, alienating themselves from their stigmatizing community, or engaging in passing techniques to manage information and their ‘discreditability’ among community.^25^

A study conducted among the survivors of Tsunami in 2004 reported that most of the children and parents suffered from 3-4 traumatic tsunami-related incidences and about 98% of the parents had a PTSD reaction. In both children and parents, the immediate subjective response to tsunami was correlated to PTSD reaction 6-8 months later. There was a significant association between children’s and parents’ exposure to the stressor and parents’ PTSD with children’s levels of post-traumatic stress reactions.^24^

Sprang and colleagues (2013) reported an increased risk of PTSD in children (30%) and parents (25%) (ID 2). This risk was higher in children and young parents. The most common diagnoses were acute stress disorder (16.7%), adjustment disorder (16.7%), and grief (16.7%). Only 6.2% of these children were diagnosed with PTSD. However, the mental health service utilization was 33.4% among quarantined families for their children, either during or after the pandemic.^13^

In study among children who were evacuated during the World War II, evacuation was not reported to be a predicator for admission to the psychiatric hospitals. Men experienced low rates of psychiatric admission between evacuated and non-evacuated siblings. For women, no association was established between evacuation and admission for a psychiatric disorder, with higher risk of mood disorders among women.^19^ A qualitative descriptive study assessed 24 children by using drawing and captions.^26^ This study suggested that Ebola was represented as a highly stigmatized and feared disease through the content of the drawings and captions. Moreover, health campaigns initiated to contain the epidemic, such as the ‘no touch’ policy and quarantine of suspected Ebola cases were the most common themes. The stigma and psychological consequences were experienced more often in children orphaned by Ebola.^26^

Elsbernd and colleagues conducted a study among nine cancer survivors that included adolescents and younger adults. The frequent challenges were both systemic and social in nature such as constraints to return to education due to symptoms and late effects, most commonly fatigue and lack of concentration. Moreover, these individuals felt that it was difficult for their peers to understand their difficulties and circumstances. In this situation, there were minimal official resources but this lack of support was compensated by family and counselors.^20^

Muenks and colleagues conducted qualitative interviews in participants who were diagnosed with MRSA skin and soft tissue infection. It was reported that 42% of survey respondents expressed that their child’s MRSA diagnosis caused a change in how household contacts interacted with one another. About 40% of caregivers stated that they personally treated their children (with a history of MRSA infection differently than their children) who had not experienced MRSA infection. The majority (91%) of participants shared their child’s MRSA diagnosis with people outside of their household.^27^

Three studies were conducted in children and adolescents in midst of the current COVID-19 pandemic. In a study by Jiao and colleagues (2020), Children aged 3-6 years were more likely than older children to manifest symptoms, such as clinginess and fear that family members could contract the infection.^23^ Other symptoms were inattention, persistent inquiry, clinging, inattention, and irritability. These distressing symptoms were relieved by using entertainment through social media and physical exercise.^23^ In a similar study conducted in Italy and Spain, the most frequent symptoms were difficulty concentrating (77%), boredom, irritability, restlessness, nervousness, feelings of loneliness, being more uneasy and increased worrying. Most parents reported a change in the emotional state and behaviors of their children. About 12% of the Italian and Spanish parents informed that family coexistence was difficult or very difficult with their children being more restless, angry and irritable. There was also increased use of screen time in both countries (82% Italian and 90% Spanish children). Spanish children stopped being physically active and were sleeping for more hours than Italian children.^22^ These symptoms were corroborated by study among children in Italy.^21^

## DISCUSSION

To our knowledge, this is the first systematic review to assess psychological impact of quarantine in children and adolescents. We identified a remarkable dearth of data on the impact of quarantine on children and adolescents during disease outbreaks. It was surprising that majority of studies we found, were for the rapidly emerging COVID-19 Pandemic rather than previous SARS or MERS outbreaks.^21^,^22^ Furthermore, none of the identified studies were designed to specifically examine children and adolescents’ own experiences or perceptions of quarantine on different aspects of their lives.

### Psychiatric Issues

Although children are vulnerable to environmental risks but statistics regarding psychological impact of home confinement, quarantine and isolation in children and adolescents are elusive and very few studies address this important aspect. Data from the COVID -19 studies from Italy, Spain and China suggests significant emotional and behavior changes during quarantine in children and adolescents.^21^–^23^ Common reactions of children and adolescents to disasters including health related ones depends on child age and developmental levels.^28^ While younger children may be clingier or regress in behaviors, older children may become more anxious, angry, restless and withdrawn while in Quarantine.^29^ Literature suggests that children often display their worries in ways that caregivers may interpret as defiant behavior.^29^ Children subjected to quarantine in pandemic disasters have more likelihood of developing acute stress disorder, adjustment disorder and grief and reported four times higher scores of PTSD compared to those who were not quarantined.^13^ The fact that high PTSD prevalence noted in literature was related to short lived infectious outbreaks like SARS, there is likelihood of huge segments of young population to experience residual and lasting distress and trauma due to larger scale and prolonged COVID-19 outbreak. It is also important to note that travel restrictions, closure or availability of limited outpatient services in many hospitals in different countries, may lead to reduce access to mental health services during the current pandemic.

Available child and adolescent evidence are consistent with broad range of impact of quarantine in adult population. Studies found elevated levels of anxiety, distress, and depression among quarantined individuals.^7^,^14^,^30^ None of the child studies looked at duration of quarantine and its association with psychological impact, but literature suggests higher PTSD symptoms in those quarantined for longer duration specifically for more than ten days.^14^ Given the prolonged quarantine and isolation in COVID-19, likelihood of worse psychological outcomes in vulnerable populations including children and adolescents won’t come as a surprise.

As there is evidence that significant burden of mental illnesses originate in young age and adult life productivity is also deeply rooted in early years, close attention to mental health of young people in quarantine is warranted to avoid any long-term consequences.^31^,^32^

### Stigma

Infectious diseases, where quarantine is required, are likely to evoke social processes that stigmatize people affected by it.^33^ Our study identified only four studies focusing on quarantine related stigma, discrimination and social exclusion felt by families and children due to Ebola, MERS, cancer and physical disability. Stigma related to quarantine and causes of quarantine has been a major theme throughout the literature, however only limited data is available regarding stigma faced by children and adolescents in this context. Children affected by HIV and AIDS were discriminated against, stigmatized and isolated by community members in a study by Khewsa et al.^34^ Similar stigmatization with reduction in social interaction with other children has been noted in relation to Ebola.^26^ Quarantined households continued to be associated with Ebola leading to secondary stigma which hampered reintegration of young people in society, long after the end of quarantine. Stigma linked with quarantine thus have real implication for children’s social relationships at community level and contribute to significant psychological distress. Political conflicts, poverty, unfounded fear of transmission of infection have all been identified as factors for perfect storm of fear and stigma.^34^

### Physical Health

Confinement during disease outbreak is likely to have negative effects on children’s physical wellbeing and it has been documented in recent COVID-19 pandemic studies as well.^22^,^23^,^35^ Parents in Italy and Spain during COVID-19 reported negative impact on physical health with less physical activity, and more screen time than usual among children.^22^ This is consistent with past evidence suggesting that children during school days are physically more active, have regular sleep patterns and less screen time, while increase screen time, less physical activities and poor dietary patterns leading to weight gain is reported during summer vacations and weekends.^35^,^36^ Spanish children showed worse behavioral and emotional response to the quarantine and one possible hypothesis proposed was permission by Italian Government for young children to go for short walk accompanied by parents and more Italian homes having gardens.

### Education

There is compelling evidence that school closure as a disease containment measure during outbreaks like Influenza can dramatically reduce the spread of disease but there may be high cost of prolonged school closures among children and adolescents.^37^ The United Nations Educational, Scientific and Cultural Organization highlighted that with mass school closures in more than 188 countries during COVID-19 Pandemic, “the global scale and speed of the current educational disruption is unparalleled”. None of the studies in our review looked at impact of quarantine on children academics and schooling. However, a recent paper by Joyce Lee highlighted the mental health effects of school closures. Some previous reviews also emphasized loss of education, nutritional problems and social isolation leading to psychological harm as few of adverse effects of school closures.^38^ Besides, academics, school routines are important for children and they access many services including mental health support through schools.^38^

Another area of concern during quarantine is increase rates of child abuse, neglect, and exploitation while children stay at home and it may go unchecked due to social isolation. Increase in reports of domestic violence in China during recent COVID-19 Pandemic is of concern. Studies from previous natural disasters, and outbreaks like the Ebola outbreak in West Africa from 2014-2016, also revealed increased rates of child abuse during disease containment measures like quarantine and isolation.^29^, ^39^ There is urgent need to monitor how prolonged school closures and strict social distancing impact children and adolescent wellbeing in the long run.

### Socialization

Social distancing measures like quarantine can worsen feelings of loneliness and isolation. Children and adolescents need to stay connected with family and friends, which gets difficult with school closures, limited visits with friends and families etc. Inability to activate your social network is noted to be associated with anxiety and distress.^40^ One of the studies in our review looked at impact of isolation on socialization of families of children with disability. They reported feeling strangers in their own communities due to rejection they faced because of their children problems. Reduction in social interaction with other children has also been reported in Ebola and HIV outbreaks.^26^,^34^ It is important to have support groups for children and families in quarantine so that they may feel connected and empowered and it can reduce psychological distress.

### Parental Perceptions

Most of the studies in our review had parents as reporter of child symptoms. Many parents isolated at home are also under lots of stress. Parental perception of quarantine impact on children and adolescents thus have element of subjectivity. Parents reporting more child emotional and behavioral difficulties were noted to be one who found family coexistence difficult or very difficult.^22^ Parental stress has been shown to predict stress reactions in children and therefore parents need to maintain their own calm. Although it is considered natural to protect children from unpleasant information, but even very young children react to environmental changes and often assume the worst. By managing their own stress better, parents can help to manage children stress.

Thus, to summarize, this review shows considerable psychological impact of quarantine and other disease containment measures among children and adolescents. Quarantine also has negative impact on their physical health, academics and social network. Many of our findings resonate strongly with adult studies calling for greater focus on quarantine related stigma experienced by children and adolescents. Psychological distress of children in Quarantine need to be considered in planning of response to any disaster including health related emergencies.

### Proposed Interventions and Components

**Fig.2:**
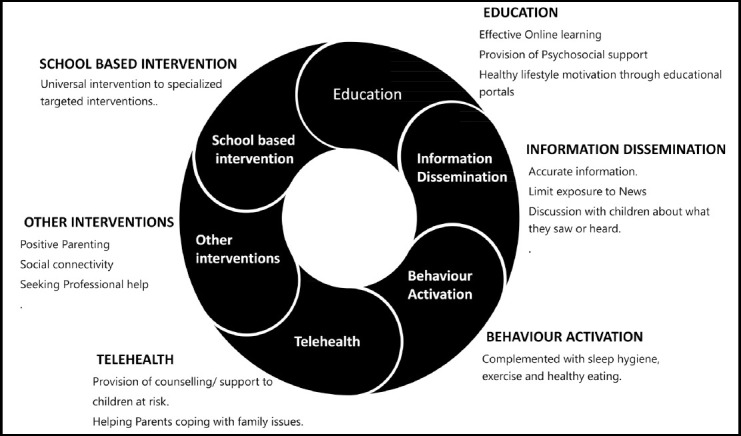
Interventions to reduce adverse psychosocial impact of quarantine in children and adolescents.

Given the evidence of adverse psychosocial impact, effective measures need to be in place to mitigate the effects of home confinement on children and adolescents. The Lancet Commission on the future of the world’s children urges various stakeholders to ensure that all children’s needs are met during these uncertain times as “early investment in children health, education and development have benefits that compound throughout the child’s lifetime, and societies as a whole”.^41^ Immediate actions are warranted in various sectors. Fig.2 provide a framework for interventions to address psychological burden and stigma among quarantined children and adolescents.

### Education

Education is one of the strongest predictors of the health of a nation and thus needs to be addressed on priority basis. With widespread and extended school closures around the globe, educational institutions need to be innovative and provide lessons and other services to students through alternate resources to minimize disruption in education. Education and health officials need to work in collaboration with public health officials and provide guidelines for effective online learning and ensuring that contents of courses meet the educational requirements. Restricting the duration of school closure to minimum and making plans and guidance documents for safe return of children in schools are also essential to prevent consequences of quarantine.

### Information dissemination from media and other sources

Kids tend to worry more, when they are kept in dark about what’s happening around them. It is a challenge to increase the sensitivity of media regarding reporting of events to reduce anxiety in the eye of lockdown and pandemics.^42^ Ensuring that children under quarantine have good, age-appropriate understanding of the illness and reason for quarantine should be a priority. Watching news with children, asking about what the child has seen or heard, providing reassurances and monitoring regularly if news is troubling or upsetting the child may help in lessening the negative effects of news during quarantine. It is important to acknowledge and validate children’s thoughts, feelings and reactions in order to provide children with emotional scaffolding they need to thrive during quarantine.

### Behavioral Activation

Behavior activation (BA) is a component of Cognitive behavior therapy that aims young people to engage more often in enjoyable activities and improve their problem-solving skills alongside addressing excess of avoidance behaviors. It can be an appropriate culturally sensitive intervention to reduce the psychological impact of quarantine among children and adolescents, complemented with other approaches like lifestyle changes, counselling and family therapy.^43^

### Healthcare system response – Telehealth

Telehealth including telepsychiatry although an established modality in developed world is yet to gain momentum and popularity in low-and-middle income countries (LMIC). It can be used as an effective tool to provide counselling and psychological support to children and adolescents at risk with prevailing higher social media use in youth.^35^ However, there needs to be some mechanism to monitor the quality of telehealth services, ensuring that ethical standards are maintained, trained professionals are providing collaborative services and appropriate referral pathways to hospitals is in place should it is required.^44^

### School-based strategies

Schools are increasingly being identified as a context in which apart from traditional subjects, life skills and “social emotional education” need to be imparted to students.^45^ This role becomes even more relevant following situations where children and adolescents are confined at home for longer periods. Many children also experience severe illness themselves or in family or loss of loved ones during infectious diseases outbreak placing them at even higher risk of psychological distress. Schools offer a unique opportunity and a cost-effective way to reach out a large number of students. In some LMIC, they could be the only mental health service provision opportunity in rural areas. WHO’s global vision of ‘health promoting school’ through multifaceted response can be helpful in post quarantine situations to prevent long term adverse consequences. 45

### Other coping strategies

### Positive Parenting

Children pick up and reacts to parental and family’s emotions and stress during quarantine. Good parental skills are extremely crucial especially, when children are quarantined at home. Quarantine can be used as a good opportunity to enhance positive interaction between parents, children and siblings, thus strengthening family bonds. Various guidelines by International organizations are available to help parents during quarantine. During this time of change and uncertainty, sticking to routines/ schedule as much as possible helps in reducing the psychological impact of quarantine.

### Social distancing, not social isolation

Social distancing measures like quarantine can worsen feelings of loneliness and isolation. Social media can play an important role in communication with others. Children and adolescents need to stay connected with family and friends virtually by phone, emails, facetime, Skype, zoom. Playing online games with friends can also be relaxing for children during quarantine

### Seeking professional help

Families should be provided information to consult mental health professionals if child is too preoccupied with illness during quarantine or exhibiting signs of severe emotional disturbances.

### Limitations of the Study

This rapid review comes with few limitations. First, meta-analysis was not performed due to different study designs, measurements tools, study outcomes, and methodology of rapid review. Second, the psychological effects should be carefully interpreted as they can be due to the effect of diasaster, disease or diasaster containment measures, or synergitic effects of both.

## CONCLUSION

Overall, this review suggests that quarantine is associated with far reaching and significant negative impact on psychological wellbeing of children and adolescents. Of more concern is the finding that this negative psychological effect can still be detected months or years later. Stigma has also been rife in children and families who underwent quarantine. As quarantine is essential to contain diseases in many cases, it is important that steps and measures are taken to make this experience less traumatic for vulnerable young people. This can be done by honest and age and developmentally appropriate communication, ensuring routines and minimizing disruption in education, encouraging healthy lifestyle, enhancing positive relationship between families, managing parental stress and incorporation of health promotion activities in school curriculum. These strategies may ensure that the physical and mental health impact of quarantine on children and adolescents are kept minimal. Further research to examine long term impact of quarantine and prolonged school closures on children are urgently needed to guide policies.

### Authors’ contributions

**NI & SN:** conceived the idea of this review article.

**SN, NI, IA, MIS, ZHB:** extracted and analyzed data, prepared tables, and wrote the manuscript.

**NI:** was responsible for the supervision of this project.

All authors approved the final version of this review article.
